# The Burden of HMPV- and Influenza-Associated Hospitalizations in Adults in New Zealand Before and After the COVID-19 Pandemic, 2012–2023

**DOI:** 10.1093/infdis/jiaf150

**Published:** 2025-07-16

**Authors:** Nayyereh Aminisani, Briony Fanslow, Timothy Wood, Lauren Jelley, Louise Thorn, Ruth Seeds, Conroy Wong, Adrian Trenholme, Cameron C Grant, Q Sue Huang

**Affiliations:** Institute of Environmental Science and Research, Wellington, New Zealand; Institute of Environmental Science and Research, Wellington, New Zealand; University of Auckland, Auckland, New Zealand; Institute of Environmental Science and Research, Wellington, New Zealand; Institute of Environmental Science and Research, Wellington, New Zealand; Institute of Environmental Science and Research, Wellington, New Zealand; Institute of Environmental Science and Research, Wellington, New Zealand; University of Auckland, Auckland, New Zealand; Department of Respiratory Medicine, Te Whatu Ora—Health New Zealand Counties Manukau, Auckland, New Zealand; Department of Paediatrics: Child & Youth Health, University of Auckland, Auckland, New Zealand; Kidz First Children's Hospital, Te Whatu Ora – Health New Zealand Counties Manukau, Auckland, New Zealand; Department of Paediatrics: Child & Youth Health, University of Auckland, Auckland, New Zealand; Starship Children's Hospital, Te Whatu Ora – Health New Zealand Te Toka Tumai Auckland, Auckland, New Zealand; Institute of Environmental Science and Research, Wellington, New Zealand

**Keywords:** human metapneumovirus, influenza, incidence, burden of disease, acute respiratory infection, adults

## Abstract

**Background:**

Understanding temporal trends of influenza and human metapneumovirus (HMPV) infections among adults and their return after the severe acute respiratory syndrome coronavirus 2 (SARS-CoV-2) (coronavirus disease 2019 [COVID-19]) pandemic is important for designing prevention and control strategies.

**Methods:**

Using the New Zealand hospital-based surveillance dataset, we compared the population-based incidence, seasonality, and clinical characteristics of influenza and HMPV infections among hospitalized adults aged 20 years and older with acute respiratory infections (ARI) in Auckland, New Zealand, from 2012 to 2023.

**Results:**

In the surveillance project, 37 185 ARI hospitalizations were detected from 2012 to 2023 among adults 20 years and older. Of the 21 649 illnesses tested for HMPV, 735 (3.4%) were positives and of the 24 138 tested for influenza, 3623 (15.0%) were positives. Crude rates of annual ARI hospitalizations per 100 000 adult residents were 9.7 (95% confidence interval [CI], 9–10.4) for HMPV and 48.2 (95% CI, 46.6–49.7) for influenza. The highest hospitalization rates for both viruses were in those aged 80 years or older, those of Māori or Pacific ethnicity, and those living in low socioeconomic status areas. There were no statistically significant differences in the HMPV-associated ARI hospitalization rates before and after the COVID-19 pandemic. In contrast, influenza-associated hospitalization rates per 100 000 were 43.0 before the pandemic, increased to 54.4 in 2022, and then 79.5 in 2023, with significant increases across most demographic groups.

**Conclusions:**

Although HMPV infections accounted for fewer ARI hospitalizations than influenza infections in all study years, relative to younger adults, HMPV-associated ARI hospitalization rates were significantly higher in older adults due to the high prevalence of underlying chronic conditions in this age group. This highlights a need for vaccine/antiviral intervention.

Influenza and human metapneumovirus (HMPV) are two leading causes of respiratory-related hospitalizations worldwide [1]. Influenza-associated hospitalization is a well-recognized feature of infections with this virus [2, 3], associated with 3–5 million cases of severe illness and 290 000 to 650 000 respiratory deaths annually, affecting 5%–10% of adults and mainly older adults [4]. Influenza infection is also a cause of serious illness for immunocompromised patients or those with chronic medical conditions [5–7]. While HMPV infection is well known to cause acute respiratory infections (ARI) in children, the burden of disease in adults, especially older adults, is less well defined [8]. Several studies have detected HMPV in 3%–6% of adults presenting to primary care practices with lower respiratory tract infections, with a higher prevalence in patients ≥65 years of age with chronic conditions [9–11]. However, published studies to date are limited by convenience sampling, short study periods, low rates of HMPV infections [12–18], and restricted data on hospitalizations associated with HMPV. Hospitalization rates reported from the United States in the pre-COVID-19 era [19, 20] were 20 per 100 000 adults aged 18–65 years and 221 per 100 000 adults aged ≥65 years. In a more recent study reporting data from 2015–2019 [21], the hospitalization rates were 36, 176, and 593 per 100 000 people for young adults aged 18–49, middle-aged adults 50–64, and older adults ≥65, respectively. Our recently published study from New Zealand [22] reported a somewhat lower crude rate of 9.8 (95% confidence interval [CI], 8.7–11.0) HMPV-associated ARI hospitalizations per 100 000 adult residents 20 years and older annually across a 4-year study period from 2012 to 2015.

In this current study, the primary objective was to examine the temporal trends related to the incidence rates, hospital outcomes, and associated comorbidities of HMPV infections compared to influenza infections among adults aged ≥20 years during an extended study period from 2012 to 2023. Our secondary objective was to understand the impact of SARS-CoV-2 (COVID-19) nonpharmaceutical interventions on HMPV and influenza infections by examining these viruses’ pre- and postpandemic epidemiology.

## METHODS

### Study Population

The Southern Hemisphere Influenza Vaccine Effectiveness Research and Surveillance (SHIVERS) project was established in 2012 and was funded by the US Centers for Disease Control and Prevention (CDC) [23]. The SHIVERS study obtained ethics approval from the Northern A Health and Disability Ethics committee (NTX/11/11/102). Funding from the US CDC finished in 2016, then the New Zealand Ministry of Health continued to fund surveillance system. SHIVERS was an active ARI surveillance project identifying patients requiring at least 1 overnight stay in the 2 main hospitals serving the central, eastern, and southern areas of Auckland, New Zealand (Auckland and Counties-Manukau District Health Boards [ADHB and CMDHB, respectively]). According to the Statistics New Zealand government census conducted in 2018, the total resident population served by these hospitals was 1 068 154, of which 779 860 people (73%) were aged ≥20 years. Of the 2018 adult catchment population, 10.2% identified as Māori (New Zealand's indigenous population), 14.4% identified as Pacific (including ethnic groups from Samoa, Cook Islands, Tonga, Niue, Fiji, Tokelau, Tuvalu, and Kiribati), 31.2% as Asian, and 44.2% as European and other ethnic groups [24].

Study nurses evaluated inpatient ARI cases through a combination of reviewing admission diagnoses and interviewing patients about their presenting signs and symptoms. Among ARI patients, they identified those who had severe acute respiratory infection (SARI), which is defined as an ARI accompanied by a fever of 38°C or higher and a cough occurring within the preceding 7 days (in 2012) or 10 days (from 2013 to 2015) [25]. To gain a better understanding of the SARI case definition and the associated disease burden, they also identified ARI cases that did not meet the SARI criteria. This included cases with either but not necessarily both cough or measured/reported fever within 10 days. A systematic sample of non-SARI patients was interviewed, and a respiratory sample was collected. In 2013, 4 non-SARI adult inpatients from each hospital were interviewed weekly during the peak winter period, which ran from 12 August to 6 October. In 2014 and 2015, this was increased to 12 non-SARI adult patients per hospital, who were interviewed weekly during a longer winter period, from late April to late September. This analysis included SARI and non-SARI samples collected not only by SHIVERS study nurses but also by clinical nurses for clinical purposes (broad conditions are described in Supplementary Material C).

There was variation in surveillance periods across ward types and years. Subject to case definition eligibility, year-round surveillance was conducted in medical wards and intensive care units (ICUs) during 2012–2015 and 2022–2023. During 2016–2021, active surveillance in ICUs was conducted year-round, while surveillance in general medical and pediatric wards was from May to October. Patients admitted with an ARI who were not tested as part of the surveillance could be tested for HMPV for clinical management purposes as ordered by caring clinicians. All SARI patients and a proportion of non-SARI patients were eligible for testing for influenza/HMPV viruses. A systematic sample of approximately 50% of non-SARI patients was also interviewed, and a respiratory sample was provided alongside those who had specimens collected for clinical purposes.

### Data Collection

Study nurses obtained verbal consent from patients, completed standardized case report forms, and collected respiratory specimens. The case report form captured patient demographics and presenting symptoms, doctor visits before hospitalisation for the acute illness event, medication usage, influenza vaccination history, comorbidities, hospital course and outcomes, epidemiological risk factors, and laboratory results for consented patients.

The Auckland and Middlemore field operation teams provided the Institute of Environmental Science and Research Health Intelligence Team with data on all SARI cases and recorded non-SARI cases through an electronic capture tool, a REDCap survey project. Laboratory test result information was also recorded electronically. Data linkage to the New Zealand Ministry of Health data collections, including hospital discharge and admission dates, ICU utilization, event diagnoses (national minimum dataset), and the national mortality collection and immunization registry during the study period, were performed for all ARI patients using each person's unique identifying number—the National Health Index.

### Laboratory Testing

Respiratory samples were tested for influenza, HMPV, and other common respiratory viruses using: (1) the US CDC real-time reverse transcriptase polymerase chain reaction (RT-PCR) protocol [26] at the National Influenza Centre, Institute of Environmental Science and Research and the ADHB laboratory; and (2) the AusDiagnostic PCR protocol [27] at the CMDHB laboratory. Further information on performance of hospital assays compared to CDC's real-time RT-PCR as a gold standard have been described in the supplementary material of our previous publication [22]. There were slight differences in testing algorithms, especially during the COVID-19 pandemic. Some samples had a full respiratory virus testing completed when the respiratory sample was collected, while other samples were first tested for SARS-CoV-2, influenza, and respiratory syncytial virus, then subjected to further testing, which included HMPV. Positivity rates reported in this study are case-based rather than sample-based; we count any positive influenza or HMPV result associated with a hospitalization event, regardless of whether the result was from a single assay or repeat test.

### Statistical Methods

Two or more ARI hospitalizations tested within 14 days were considered the same episode. Annual average ARI hospitalization rates were calculated and stratified by age, sex, prioritized ethnic groups (Māori, Pacific, Asian, or European and other ethnicity), and socioeconomic status (SES) as estimated by the 2013 and 2018 New Zealand Deprivation Index, a small-area composite measure where 1 indicates the individual was living in a household in the least socioeconomically deprived quintile [28]. The area-level New Zealand Deprivation Index was used to separate the study population into SES quintile categories. Incidence rates were calculated by dividing the number of HMPV- or influenza-associated ARI hospitalizations (singular episodes) by the number of adults residing in the study area.

Both crude and adjusted incidence rate ratios (IRRs) for each virus were determined; adjusting for age, sex, and ethnic group using Poisson regression models. Due to the onset of the COVID-19 pandemic, very few HMPV cases and zero influenza cases were detected in 2020 and 2021. To maintain consistency, the pandemic years (2020 and 2021) were excluded from the Poisson models, enabling analysis across the more consistent years before (2012–2019) and after the pandemic (2022–2023). The proportion of SARI and non-SARI patients tested for influenza and HMPV is provided in Supplementary Table 2.

The correction for nontesting for HMPV and influenza among ARI patients was done using the multiple imputation by chained equations (MICE) method [29]. Before imputation for missing outcome data, we verified that nontested patients were missing at random, using a missing completely at random test (MCAR) [30]. Then, we createdg 50 imputed datasets of HMPV and influenza results in which age, ethnicity, SES, ARI type (SARI case definition), sex, and week of hospitalization included as predictors of missingness. Weekly counts of ARI hospitalizations among people aged 20 years and over, along with HMPV and influenza virus detections, are displayed using noncorrected test results.

To compare the clinical characteristics, we restricted our analysis to patients with positive results for HMPV or influenza. We compared underlying conditions and clinical outcomes, including hospital length of stay (LOS), ICU admission, and ICU LOS between HMPV and influenza-associated hospitalizations. A small number (n = 24) of cases positive for both viruses were excluded from the analysis; n = 15 were <50 years of age, 11 of them had 2 or more comorbid conditions, and 2 of them required ICU admission. Influenza vaccination was presented as a dummy variable (yes/no) using both self-reported and linked data from the immunization registry. We calculated the Charlson Comorbidity Index (CCI) [31] and also presented selected chronic medical conditions such as chronic respiratory disease (asthma and chronic obstructive pulmonary disease [COPD]), cardiovascular conditions, congestive heart failure (CHF), and diabetes using the International Statistical Classification of Diseases and Related Health Problems, Tenth Revision (ICD-10) codes. We compared the HMPV and influenza-associated hospitalizations according to the severity of comorbidities. The CCI assigns weights of 1, 2, 3, and 6 to each of the existing comorbidities whose final score is obtained by the sum of these weights. We chose to use the age-adjusted CCI in which the sum of the weights of the comorbidities were adjusted for age groups: 50 to 59 years old (1 point), 60 to 69 years old (2 points), 70 to 79 years old (3 points), and ≥80 years (4 points) [32]. We also presented the modification of the CCI scoring introduced by Quan et al [33]. (Supplementary Material B).

For the clinical outcomes analysis, categorical variables were analyzed with χ^2^ tests and continuous variables using the *t* test, a 1-way analysis of variance. We used quantile regression to test the equality of medians for LOS and days of stay in the ICU. All analyses were performed using STATA 18.

## RESULTS

### Study Population

There were 37 185 overnight ARI admissions to study hospitals among adults from 2012 to 2023 (Supplementary Figure 1). Of these admissions, 21 649 (58.2%) were tested for HMPV, and 24 138 (64.9%) were tested for influenza. Testing rates for the SARI and non-SARI patient groups were variable over the years, and by virus (presented in Supplementary Material A Table 2). Of those tested, 735 (3.4%) were positive for HMPV, and 3623 (15.0%) were positive for influenza. Excluding the 2 years during the COVID-19 pandemic when little (HMPV) to no (influenza) transmission was detected, total positivity was 3.7% for HMPV and 16.8% for influenza.

Overall, and subgroup-specific average annual ARI hospitalization rates associated with HMPV were lower than those for influenza, regardless of whether imputed values for nontested cases were included in the analysis or not (Table 1). The nonimputed average annual hospitalization rate was 9.7 (95% CI, 9.0–10.4) for HMPV and 48.2 (95% CI, 46.6–49.7) for influenza per 100 000 residents. After imputing results for nontested cases, the estimated HMPV and influenza hospitalization rates were 15.2 (95% CI, 14.3–16.1) and 70.1 (95% CI, 68.2–72.0) per 100 000 residents, respectively (Table 1).

**Table 1.** Acute Respiratory Hospitalization Rates Associated With HMPV or Influenza Among Adults by Demographic Characteristic, Crude and Corrected for Nontesting, Auckland, New Zealand 2012–2023

Characteristics	HMPV-Associated Hospitalization Rates per 100 000		Influenza-Associated Hospitalization Rates per 100 000	
	Crude, Rate (95% CI)	Imputed for Nontesting,^a^ Rate (95% CI)	Crude, Rate (95% CI)	Imputed for Nontesting,^a^ Rate (95% CI)
Overall	9.7 (9.0–10.4)	15.2 (14.3–16.1)	48.2 (46.6–49.7)	70.1 (68.2–72.0)
Year^b^				
2012	9.8 (7.5–12.2)	19.3 (16.0–22.6)	45.6 (40.5–50.6)	91.7 (84.5–98.8)
2013	9.1 (6.9–11.4)	18.5 (15.3–21.6)	29.0 (25.0–33.0)	90.2 (83.1–97.2)
2014	12.9 (10.3–15.6)	18.9 (15.7–22.1)	61.8 (56.0–67.5)	87.6 (80.7–94.5)
2015	12.0 (9.4–14.5)	17.6 (14.6–20.7)	45.3 (40.4–50.2)	68.6 (62.6–74.6)
2016	9.6 (7.4–11.9)	14.6 (11.9–17.4)	24.5 (20.9–28.0)	47.9 (42.8–52.9)
2017	6.2 (4.4–7.9)	8.0 (6.0–10.0)	51.4 (46.4–56.5)	63.7 (58.1–69.3)
2018	9.0 (6.9–11.1)	10.6 (8.3–12.9)	32.6 (28.6–36.6)	40.3 (35.8–44.7)
2019	8.1 (6.1–10.0)	9.8 (7.6–12.0)	53.5 (48.4–58.6)	60.7 (55.3–66.2)
2022	8.8 (6.8–10.9)	16.1 (13.4–18.9)	54.4 (49.3–59.5)	70.3 (64.5–76.1)
2023	11.7 (9.3–14.0)	19.5 (16.5–22.6)	79.5 (73.4–85.7)	84.7 (78.3–91.0)
Age, y				
20–49	3.4 (2.9–4.0)	4.9 (4.3–5.6)	24.4 (23.0–25.8)	33.3 (31.7–35.0)
50–64	9.9 (8.5–11.4)	15.0 (13.2–16.8)	52.5 (49.1–55.9)	74.0 (70.0–78.1)
65–79	27.8 (24.4–31.2)	44.3 (40.0–48.6)	103.0 (96.4–109.6)	153.1 (145.0–161.1)
80+	52.8 (44.2–61.4)	92.0 (80.6–103.3)	237.2 (218.9–255.4)	386.4 (363.2–409.7)
Sex				
Female	11.6 (10.5–12.6)	18.0 (16.6–19.3)	53.4 (51.1–55.7)	77.0 (74.3–79.8)
Male	7.7 (6.8–8.6)	12.2 (11.1–13.3)	42.6 (40.5–44.7)	62.7 (60.1–65.3)
Ethnicity				
European and other	9.4 (8.4–10.4)	15.1 (13.8–16.3)	38.3 (36.3–40.4)	58.3 (55.8–60.8)
Māori	11.1 (8.6–13.5)	17.6 (14.6–20.7)	73.8 (67.5–80.1)	106.2 (98.6–113.8)
Pacific	19.9 (17.4–22.5)	30.5 (27.4–33.7)	107.7 (101.8–113.7)	151.4 (144.4–158.5)
Asian	3.9 (3.1–4.8)	5.9 (4.9–7.0)	22.7 (20.6–24.7)	32.2 (29.8–34.7)
SES^c^				
1, highest quintile	5.3 (4.0–6.6)	8.6 (7.0–10.2)	25.2 (22.5–28.0)	37.9 (34.5–41.3)
2	8.2 (6.7–9.7)	13.2 (11.3–15.0)	35.8 (32.8–38.9)	53.5 (49.8–57.3)
3	8.0 (6.5–9.5)	12.6 (10.7–14.4)	36.2 (33.1–39.4)	53.6 (49.8–57.4)
4	9.7 (7.9–11.5)	15.1 (12.9–17.3)	53.0 (48.9–57.1)	75.2 (70.3–80.1)
5, lowest quintile	14.4 (12.8–16.0)	22.3 (20.3–24.3)	75.5 (71.9–79.2)	108.8 (104.4–113.3)

Abbreviations: CI, confidence interval; HMPV, human metapneumovirus; SES, socioeconomic status.

^a^Correction for nontesting among patients with acute respiratory infections has been done using the multiple imputation by chained equations (MICE) method of imputation in STATA.

^b^Years 2020 and 2021 were not included in the model.

^c^SES based on small area-level measure of household deprivation derived from the national census (New Zealand Deprivation Index 2013, 2018) where 1 indicates the individual is living in a household that is in the least socioeconomically deprived quintile.

Age was an independent risk factor for both viruses, as evidenced in the fully adjusted Poisson regression model (Table 2). Even without imputation for missing testing results, patients who were ≥ 80 years old were 18.5 times more likely to be HMPV positive than those aged 20–49 years (IRR, 18.5; 95% CI, 14.7–23.4). In comparison, patients who were ≥ 80 years old were 12.8 time more likely to be influenza positive (IRR, 12.8; 95% CI, 11.6–14.2) than patients aged 20–49 years. The same relative pattern was observed when the imputed data were modelled (Table 2).

**Table 2.** Adjusted Incidence Rate Ratio of Acute Respiratory Hospitalisations Associated With HMPV or Influenza Among Adults Aged 20 Years or Older by Demographic Characteristics not Corrected and Corrected for Non-testing, Auckland, New Zealand 2012–2023

Characteristics	Adjusted Rate Ratio, Crude		Adjusted Rate Ratio, Imputed for Nontesting^a^	
	HMPV, IRR (95% CI)	Influenza, IRR (95% CI)	HMPV, IRR (95% CI)	Influenza, IRR (95% CI)
Age, y				
20–49	Ref	Ref	Ref	Ref
50–64	3.2 (2.5–3.9)	2.4 (2.2–2.6)	3.3 (2.8–4.0)	2.5 (2.3–2.7)
65–79	9.4 (7.7–11.5)	5.1 (4.6–5.5)	10.5 (8.9–12.3)	5.6 (5.2–6.0)
80+	18.5 (14.7–23.4)	12.8 (11.6–14.2)	22.4 (18.6–27.0)	15.5 (14.3–16.9)
Sex				
Female	Ref	Ref	Ref	Ref
Male	0.7 (.6–.8)	0.8 (.8–.9)	0.7 (.7–.8)	0.9 (.8–.9)
Ethnicity				
European/other	Ref	Ref	Ref	Ref
Māori	1.7 (1.3–2.2)	2.4 (2.1–2.6)	1.8 (1.4–2.2)	2.5 (2.3–2.7)
Pacific	2.6 (2.1–3.2)	3.1 (2.9–3.4)	2.6 (2.2–3.1)	3.1 (2.9–3.4)
Asian	0.6 (.5–.8)	0.8 (.7–.9)	0.6 (.5–.7)	0.8 (.7–.9)
SES^b^				
1, highest quintile	Ref	Ref	Ref	Ref
2	1.6 (1.2–2.2)	1.4 (1.2–1.6)	1.6 (1.3–2.0)	1.4 (1.3–1.6)
3	1.6 (1.2–2.2)	1.4 (1.2–1.6)	1.6 (1.2–2.0)	1.4 (1.3–1.6)
4	1.9 (1.4–2.5)	1.8 (1.6–2.1)	1.8 (1.4–2.3)	1.8 (1.6–2.0)
5, lowest quintile	2.2 (1.7–2.9)	2.0 (1.8–2.3)	2.1 (1.7–2.7)	2.0 (1.8–2.2)

Hospitalization rates have been adjusted for year (excluding 2020, 2021), age, sex, ethnicity and SES.

Abbreviations: CI, confidence interval; IRR, incidence rate ratio; Ref, reference; SES, socioeconomic status.

^a^Correction for nontesting among patients with acute respiratory infections has been done using the multiple imputation by chained equations (MICE) method of imputation in STATA.

^b^SES based on small area-level measure of household deprivation derived from the national census (New Zealand Deprivation Index 2013, 2018), where 1 indicates the individual is living in a household that is in the least socioeconomically deprived quintile.

Other independent factors associated with HMPV and with influenza infection were ethnic group and SES. HMPV and influenza had similar hospitalization patterns across ethnic groups, with higher rates among Pacific and Māori ethnic groups and lower rates among the Asian ethnic group. Hospitalization rates were higher in those living in neighborhoods with lower SES. The results follow the same pattern in a model using the imputed data. We also observed that HMPV (IRR, 0.7; 95% CI, .6–.8) and influenza (IRR, 0.8; 95% CI, .8–.9) hospitalization rates were lower in men than women (Table 2). We found that women had a higher rate of hospitalizations for both viruses.

### Seasonal Trends in HMPV and Influenza Acute Respiratory Infection Hospitalizations

**Figure 1.**
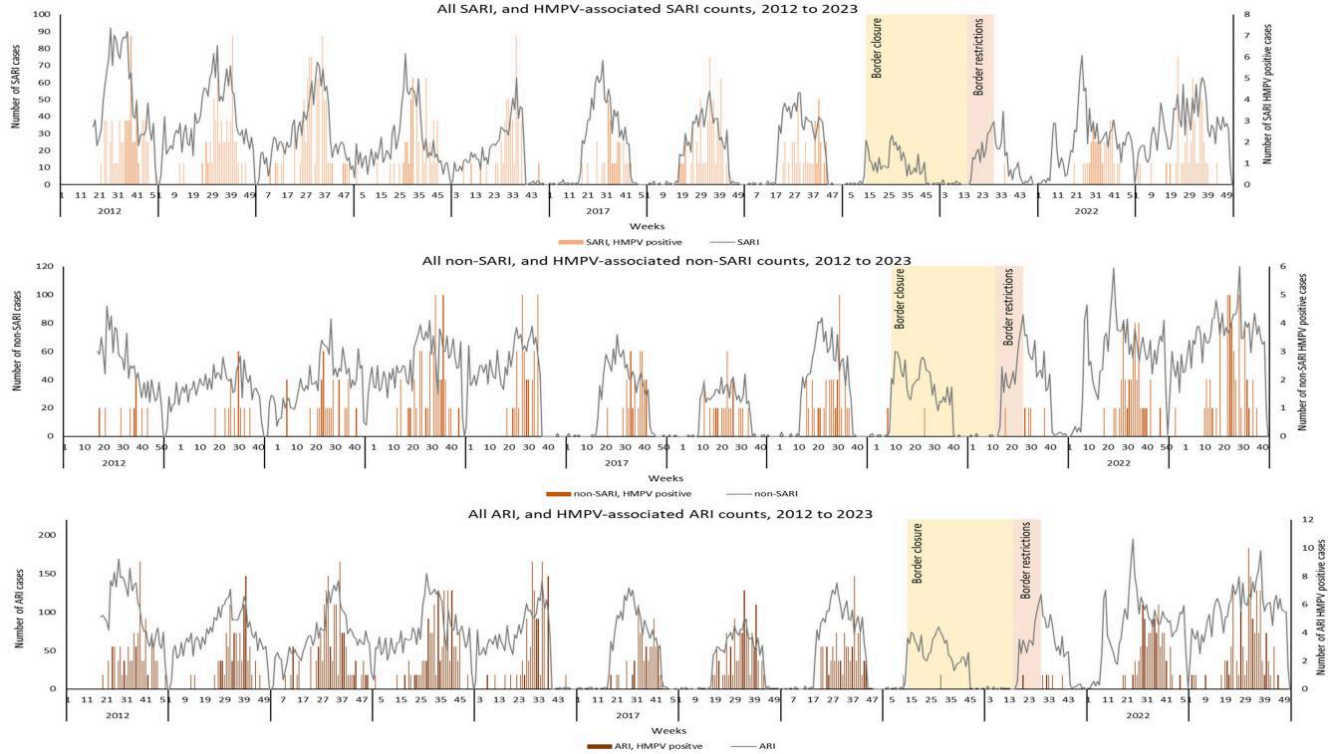
Weekly counts of acute respiratory hospitalizations (ARI) among adults aged 20 years and older, overall and HMPV associated, Auckland, New Zealand 2012–2023. There was seasonal surveillance in place for SARI during 2016–2019. Abbreviations: ARI, acute respiratory infection; HMPV, human metapneumovirus; SARI, severe acute respiratory infection.

**Figure 2.**
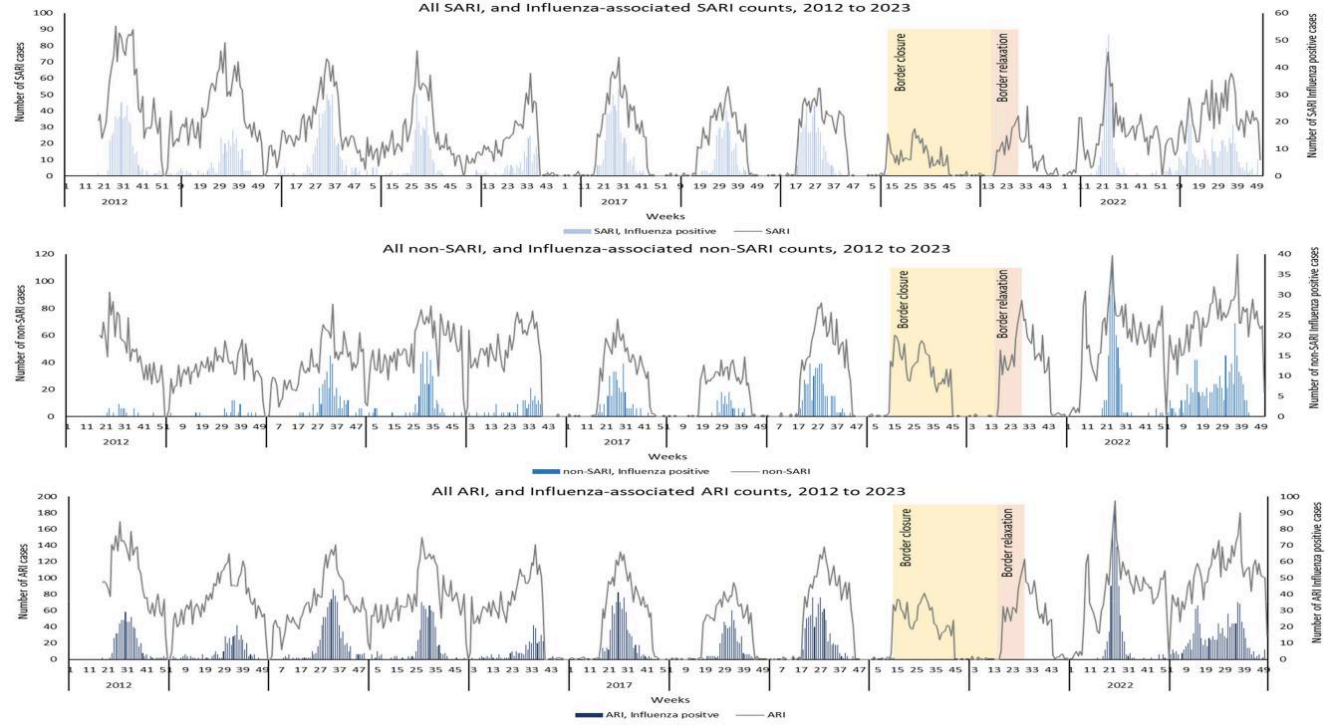
Weekly counts of acute respiratory hospitalizations (ARI) among adults aged 20 years and older, overall and influenza associated, Auckland, New Zealand 2012–2023. There was the seasonal surveillance in place for SARI during 2016–2019. Abbreviations: ARI, acute respiratory infection; HMPV, human metapneumovirus; SARI, severe acute respiratory infection.

There was a clear seasonal pattern for influenza hospitalizations among adults each year, with hospitalizations primarily detected during the southern hemisphere winter months (June to August). The HMPV season coincided with influenza, but the HMPV seasons tended to be lengthier, lasting to late October. However, this pattern was disrupted after the onset of the COVID-19 pandemic, and very few HMPV cases were detected in 2020 and 2021. In 2022, HMPV infections displayed a more rapid rise and fall, albeit with a delayed peak compared to influenza. In the following year, 2023, the pattern resembled the prepandemic scenario (Figure 1). As shown in Figure 2, no influenza cases were detected in 2020 and 2021. In 2022, influenza infections also exhibited a more rapid rise and decline, with a higher peak rate occurring in week 22 (first week in July) and sharply decreasing in week 29. The following year, 2023, the pattern differed, with notably more cases across and towards the end of the season.

### Hospitalization Rates Before and After the COVID-19 Pandemic

HMPV-associated hospitalization rates were lower than those for influenza, both in the 8 years before the COVID-19 pandemic (2012–2019) and the 2 years following (2022 and 2023). In all years, HMPV- and influenza-associated hospitalization rates were higher among women, older adults, people of Māori and Pacific ethnic groups, and people residing in more socioeconomically deprived neighborhoods (Table 3).

**Table 3.** Comparison of the Epidemiological Pattern of ARI Hospitalizations Associated With HMPV and Influenza Before and After COVID-19 in New Zealand

Characteristic	2012–2019		2022		2023	
	IR^a^ (95% CI)	2020–2021	IR (95% CI)	Changes in IR^b^ 2022 vs 2012–2019	IR (95% CI)	Changes in IR^a^ 2023 vs 2012–2019
HMPV						
Total,^a^ No.	9.70 (8.90–10.50)	…	8.8 (6.8–10.9)	0.9 (.7–1.1)	11.7 (9.3–14)	1.1 (.9–1.4)
Sex						
Female	11.4 (10.2–12.6)	…	9.6 (6.6–12.6)	0.8 (.6–1.1)	14.5 (10.8–18.1)	1.2 (.9–1.5)
Male	7.4 (6.4–8.4)	…	8.1 (5.3–10.9)	1 (.7–1.5)	8.8 (5.9–11.8)	1.1 (.8–1.6)
Age group, y						
20–49	3.2 (2.7–3.8)	…	3.1 (1.6–4.7)	1 (.6–1.7)	5.1 (3–7.1)	1.6 (1–2.5)
50–64	9.6 (8–11.3)	…	11.6 (6.8–16.5)	1.2 (.7–1.8)	10.5 (5.9–15.2)	1 (.7–1.7)
65–79	28.3 (24.4–32.3)	…	22.7 (13.6–31.8)	0.8 (.5–1.2)	29.4 (19.2–39.5)	1 (.7–1.5)
80+	55.5 (45.4–65.5)	…	31.8 (12.1–51.5)	0.6 (.3–1.1)	56 (30.1–81.9)	1 (.6–1.6)
Ethnicity						
European/other	9 (7.9–10.1)	…	8.3 (5.2–11.3)	0.8 (.6–1.2)	13.5 (9.6–17.5)	1.3 (1–1.8)
Māori	11.6 (8.7–14.4)	…	10.3 (3.6–17.1)	0.8 (.4–1.7)	9 (2.8–15.2)	0.7 (.3–1.5)
Pacific	19.8 (17–22.7)	…	17.3 (10.1–24.6)	0.8 (.5–1.3)	23.1 (14.8–31.3)	1.1 (.7–1.6)
Asian	3.8 (2.8–4.7)	…	4.8 (2.1–7.5)	1.2 (.6–2.2)	4.4 (1.8–6.9)	1.2 (.6–2.2)
SES						
1, least deprived	5 (3.6–6.3)	…	4.6 (.9–8.3)	0.9 (.4–2.1)	8.5 (3.5–13.6)	1.6 (.9–3.2)
2	8.2 (6.5–9.8)	…	7.1 (2.9–11.3)	0.8 (.4–1.5)	9.7 (4.8–14.7)	1.1 (.6–1.8)
3	7.8 (6.1–9.4)	…	7.4 (3.2–11.7)	0.9 (.5–1.6)	10 (5.1–14.9)	1.2 (.7–2)
4	9.4 (7.4–11.4)	…	10.2 (5–15.4)	1.1 (.6–1.9)	10.9 (5.5–16.2)	1.1 (.7–1.9)
5, most deprived	14.3 (12.5–16.1)	…	12.8 (8–17.6)	0.8 (.6–1.2)	16.8 (11.3–22.3)	1.1 (.8–1.5)
Influenza						
Total,^a^ No.	43 (41.4–44.7)	…	54.4 (49.3–59.5)	1.2 (1.1–1.3)	79.5 (73.4–85.7)	1.7 (1.6–1.9)
Sex						
Female	47.1 (44.7–49.5)	…	65.6 (57.8–73.5)	1.3 (1.1–1.5)	88.2 (79.1–97.3)	1.7 (1.5–1.9)
Male	38.6 (36.4–40.9)	…	42.9 (36.4–49.3)	1 (.9–1.2)	70.6 (62.4–78.9)	1.7 (1.5–1.9)
Age group, y						
20–49	21.4 (19.9–22.9)	…	27.8 (23.1–32.5)	1.3 (1.1–1.6)	44.1 (38.1–50.1)	2.1 (1.8–2.4)
50–64	47.7 (44–51.4)	…	51.7 (41.5–61.9)	1 (.8–1.3)	88 (74.6–101.3)	1.8 (1.5–2.1)
65–79	91.1 (84–98.2)	…	130.7 (108.9–152.5)	1.4 (1.2–1.7)	151.3 (128.2–174.4)	1.6 (1.4–1.9)
80+	229 (208.6–249.5)	…	219.3 (167.6–271.1)	0.9 (.7–1.2)	307.9 (247.3–368.6)	1.3 (1–1.6)
Ethnicity						
European/other	37.5 (35.2–39.7)	…	29.6 (23.8–35.4)	0.7 (.6–.9)	54.7 (46.8–62.6)	1.4 (1.2–1.6)
Māori	58.4 (51.9–64.8)	…	128.5 (104.7–152.3)	2.1 (1.7–2.6)	113.2 (91.1–135.3)	1.8 (1.5–2.3)
Pacific	89.5 (83.4–95.7)	…	147.2 (126.1–168.3)	1.6 (1.3–1.8)	196.7 (172.6–220.8)	2.1 (1.8–2.4)
Asian	21.1 (18.8–23.3)	…	15.5 (10.6–20.3)	0.7 (.5–1)	40 (32.2–47.8)	1.7 (1.4–2.2)
SES						
1, least deprived	23.9 (20.9–26.9)	…	20 (12.3–27.7)	0.8 (.5–1.2)	41.1 (30.1–52.2)	1.6 (1.2–2.2)
2	32.7 (29.4–36)	…	37.4 (27.8–47.1)	1.1 (.8–1.4)	57.8 (45.8–69.8)	1.7 (1.3–2.1)
3	34.9 (31.4–38.3)	…	27.9 (19.8–36.1)	0.8 (.6–1)	54.2 (42.8–65.6)	1.5 (1.1–1.8)
4	47.9 (43.4–52.4)	…	53 (41.2–64.8)	1.1 (.8–1.3)	84.2 (69.4–99)	1.7 (1.4–2)
5, most deprived	64.1 (60.3–67.9)	…	109.1 (95.1–123.2)	1.6 (1.4–1.8)	134 (118.5–149.5)	1.9 (1.7–2.2)

Abbreviations: CI, confidence interval; HMPV, human metapneumovirus; IR, incidence rate; SES, socioeconomic status.

^a^IRs were calculated by dividing the number of HMPV/influenza-associated acute respiratory infection hospitalizations by the number of people residing in the study area (residents of Auckland or Counties-Manukau District Health Boards hospital catchment areas during the study period).

^b^IRs in 2022 and 2023 were compared to their values in 2012–2019 as a reference year adjusted for age, sex, ethnicity, and SES based on a small area-level measure of household deprivation derived from the New Zealand national census (New Zealand Deprivation Index 2013, 2018) where 1 indicates the individual is living in a household that is in the least socioeconomically deprived quintile and 5 indicates an individual living in a household that is in the most socioeconomically deprived quintile of all New Zealand households.

While there were no statistically significant differences in the IRs (per 100 000 residents) for HMPV-associated ARIs among adults before and after the COVID-19 pandemic, there were some noteworthy increases (Table 3). The rate increased from 8.8 in 2022 to 11.7 in 2023, compared to an average prepandemic rate of 9.7. For women, the rate rose to 14.5 in 2023, from 11.4 in the prepandemic years. Among younger adults aged 20–49 years, the rate increased from 3.2 prepandemic to 5.1 in 2023.

The influenza-associated rate (per 100 000 residents) among adults was 43.0 before the pandemic (2012–2019) and rose to 54.4 in 2022 and 79.5 in 2023, with significant increases across most demographic groups (Table 3). In 2022, the greatest rise in hospitalization rates was observed for those aged 65–79 years, to 130.7 from 91.1 prepandemic. Increases were observed in 2023 for all adults compared to prepandemic levels, particularly among younger adults for whom hospitalization rates doubled. For women, rates increased to 66 in 2022 and 88 in 2023, compared with prepandemic average rates of 47, while rates for men did not increase in 2022 but rose to 70.6 in 2023. In both years after the pandemic, the hospitalization rates doubled among Māori, being 128.5 in 2022, and remained higher in 2023 than the prepandemic rate of 58.4. Pacific peoples experienced a significant increase, rising to 196.7 by 2023 (more than double the prepandemic rate of 89.5). In 2022, there was a notable increase in influenza-associated hospitalization rates among those residing in the most deprived neighborhoods (109.1 in 2022 compared to 64.0 in 2012–2019), followed by an increase in rates across all SES quintiles in 2023.

### Clinical Characteristics of HMPV and Influenza Associated Hospitalizations Among Patients With Positive Results for HMPV or Influenza

Clinical information was not available for all positive patients. We compared the clinical characteristics of 709 HMPV-positive and 3581 influenza-positive cases whose clinical data were available. (Table 4).

**Table 4.** Comparison of Characteristics of HMPV or Influenza Confirmed Acute Respiratory Infection Hospitalizations Auckland, New Zealand 2012–2023

Clinical Outcomes	HMPV (n = 709)	Influenza (n = 3581)	*P* Value
Stay in hospital, d, mean (±SD)	4.7 (±4.1)	3.9 (±3.8)	< .001
Stay in hospital, d, median (IQR)	3 (2–6)	3 (1–5)	>.99^a^
ICU admission	23 (3.2)	147 (4.1)	.283
Stay in ICU, d, mean (±SD)	4.1 (3.9)	5.9 (6.4)	.208
Stay in ICU, d, median (IQR)	2.5 (1.5–6.5)	3.0 (1.7–9.6)	.734^a^
Death <30 d	13 (1.8)	91 (2.5)	.263
Influenza vaccination	213 (30.0)	748 (20.9)	<.001
Selected chronic medical conditions^b^			
Chronic respiratory disease	202 (28.5)	726 (20.3)	<.001
Cardiovascular, total^c^	222 (31.3)	978 (27.3)	.03
Diabetes	234 (33.0)	1089 (30.4)	.172
Congestive heart failure	153 (21.6)	630 (17.6)	.012
Severity of comorbidities^d^			
Age-adjusted CCI			
No risk	179 (25.3)	1225 (34.2)	
Low or moderate risk	250 (35.2)	1087 (30.4)	<.001
High risk	280 (39.5)	1269 (35.4)	
Updated CCI			
No risk	207 (29.2)	1374 (38.4)	
Low or moderate risk	456 (64.3)	1979 (55.3)	<.001
High risk	46 (6.5)	228 (6.4)	

Data are No. (%) except where indicated.

Abbreviations: CCI, Charlson Comorbidity Index; HMPV, human metapneumovirus; ICD-10, International Classification of Diseases-Tenth Revision;

ICU, intensive care unit; IQR, interquartile range.

^a^Quantile regression was used for the equality of medians.

^b^According to ICD-10 codes.

^c^Including cerebrovascular coronary artery disease.

^d^The CCI compiles ICD codes for diseases such as myocardial infarction, congestive heart failure, peripheral vascular disease, cerebrovascular disease, pulmonary diseases, rheumatic disease, dementia, hemiplegia, diabetes, chronic kidney disease, liver disease, peptic ulcer disease, cancer, and HIV/AIDS. For age-adjusted CCI and updated CCI, no risk indicates no comorbid burden, low or moderate risk indicates ≥ 1 to ≤ 4 comorbidities, and high risk indicates ≥ 5 comorbidities.

Chronic respiratory disease (*P* value < .001) and cardiovascular disease (combined cardiovascular diseases and cerebrovascular accidents; *P* value = .030) were significantly more common among those patients with HMPV-associated hospitalizations than those with influenza-associated hospitalizations. Frequency of CHF was also higher in patients with HMPV compared with those with influenza infections (*P* value = .012; Table 4). In general, HMPV-positive patients reported having more comorbidities than those testing positive for influenza (71.4% vs 64.5%, *P* value < .001). They were more frequently classified as medium or high risk when applying Charlson comorbidity scores (Table 4). The proportion requiring ICU admission did not differ (*P* value = .283). Moreover, the median ICU LOS for adults with influenza was nearly half a day longer in ICU than for adults with HMPV; athough it was not statistically significant (3.0 days [IQR:1.7–9.6 days] vs 2.5 days [IQR:1.5–6.5 days], respectively, *P* value = .734). Although HMPV-positive patients had, on average, longer LOS than influenza-positive individuals (*P* value <.001), the median LOS was 3 days (IQR:2–6 days) for HMPV-positive hospitalizations versus 3 days (IQR:1–5 days) for influenza-positive hospitalizations, and it was not statistically significant (*P* value > .99). Death within 30 days of admission was not significantly different between the 2 groups (13 [1.8%] vs 91 [2.5%]; *P* value = .263). People with HMPV infections were more likely to have received the influenza vaccination (*P* value <.001).

## DISCUSSION

This article compares the disease burden of laboratory-confirmed HMPV infections with laboratory-confirmed influenza infections over 12 study years using active, integrated, population-based surveillance in patients hospitalized with ARI. Routinely collected administrative data provided clinical information on HMPV- and influenza-associated hospitalizations. In all years, rates of hospitalization associated with both viruses were generally higher among women, older adults, people of Māori and Pacific ethnic groups, and people residing in more socioeconomically deprived neighborhoods.

Although HMPV-associated hospitalization rates in adults aged ≥20 years during 2012–2023 were lower compared to influenza hospitalization rates, the differences between hospitalization rates for young and older people were more pronounced for HMPV than for influenza. The HMPV season typically was longer than the influenza season, with an observed delay of onset by about 2 months. This delay may indicate the need to extend the surveillance period to capture a larger proportion of HMPV infections.

The COVID-19 pandemic disrupted annual seasonal patterns; due to the implementation of border closures and isolation measures [34], transmission was prevented, resulting in very few HMPV and no influenza cases in 2020 and 2021 [35]. In 2022, HMPV infections rose and fell quickly, with a delayed peak compared to influenza. By 2023, the trend resembled prepandemic years. In 2022, influenza infections peaked rapidly. In 2023, however, there was a noticeable increase in influenza hospitalizations toward the end of the season. While increased viral detection rates could be in part due to more overall testing in 2022 and 2023 after the COVID-19 pandemic compared to some prior years (Supplementary Table 2), similar increases have been observed globally and characterized as a response to a population immunity gap [35–37]. After the COVID-19 pandemic, changes in demographic patterns of HMPV- and influenza-associated hospitalizations (IRs) were observed. These changes potentially signal an epidemiological shift in the distribution of infections across population subgroups defined by age, ethnicity, and SES. While the degree and nature of these changes might differ between HMPV and influenza, factors such as differences in immunity gap, virus type/subtype prevalence, contact rates, and health-seeking behavior among these subpopulations may influence the observed patterns and present further research opportunities. Moreover, the HMPV virus potentially has a different evolution rate and slower mutation rates than influenza. HMPV mutates and changes over time, with new strains emerging. However, the changes are gradual and based on previously circulating strains, resulting in a high level of immunity in the population [38]. Thus, preexisting immunity to HMPV from the prior season can protect people more effectively than the influenza virus. This would narrow down the extent of seasonal variations for HMPV compared to influenza. In the case of influenza, a new variant of the virus can evolve and enter the human population by combining a human version with an animal version of the virus from what is known as the animal reservoir. They can evade existing immunity and gain a competitive advantage through surface protein mutations that produce new antigenic variants [39]. There is no such animal reservoir of related viruses known for HMPV. We do not know the mechanism behind the observed differential burden between influenza and HMPV. This variation may be due to differences in the evolutionary rates of the 2 viruses or the distinct strategies they use to evade immune responses.

Our study found a crude positivity rate of 3.7% for HMPV and 16.8% for influenza during 2012–2023 and the imputed HMPV and influenza hospitalization rates (15.2 and 70.1 per 100 000 residents, respectively) were similar to our previous report [22]. However, HMPV-associated hospitalizations were lower in our study than those from similar studies using hospital surveillance [19, 20]. We addressed in our previous study [22] the possible explanations for the higher reported rates in those studies compared to our study, including small sample size in other studies, study periods of only 1 year during the novel influenza A H1N1 pandemic [20], and missing cases due to seasonal surveillance and not year-round surveillance [19].

Using statewide hospital discharge data recently, Zimmerman et al [21] estimated the population-based hospitalization burden of common respiratory viruses, including HMPV, over 4 years. They found hospitalization rates of 36, 176, and 593 per 100 000 people for young adults aged 18–49 years, middle-aged adults 50–64 years, and older adults ≥65 years retrospectively, which again were higher than those we report in the current analysis. Another possible explanation for the higher estimates in the other studies is their approach to account for missingness, which was managed by multiplying the proportion of enrolled patients positive for HMPV/influenza by the total number of residents with an ICD-classified ARI hospitalization during influenza seasons. In contrast, we used a multiple imputation method.

In our study hospitalizations for HMPV increased with age, particularly in those aged 65 and older, where the rate was higher than that of influenza. This trend aligns with previous studies [19, 20, 22] indicating that HMPV significantly impacts health at the extremes of age, more so than for influenza, partly due to higher vaccination rates for influenza among older adults. This highlighted a need for the development of HMPV vaccines for this vulnerable age group.

Women experience higher hospitalization rates for both viruses, but there is limited prospective human research. Animal studies show that adult female mice have greater inflammation and immune responses to H1N1 and H3N2, resulting in more severe outcomes than males [40]. In males, androgens help protect against severe influenza A virus infections by suppressing inflammation [41]. Epidemiological studies indicate that women of reproductive age have higher rates of influenza and related hospitalizations than men, although this trend reverses before puberty and in older populations [42]. While pregnancy increases the risk of severe influenza outcomes for women, it does not explain all cases [43]. Previous studies showed a female predominance in the hospitalization rate due to HMPV [11, 19] but the mechanism has not clearly been addressed.

Little is known about HMPV hospitalization rates by ethnic group or SES among adults. The present study found a significant trend of increasing HMPV-associated hospitalization rates with increasing SES disadvantage, similar to our previous publication [22]. We also found that people of Māori or Pacific ethnic groups showed substantially higher hospitalization rates than people of European and other ethnicities. The same pattern was observed for influenza-associated hospitalization rates and was consistent with our previous research and recent studies demonstrating disproportionate hospitalization rates according to sociodemographic factors [22, 44–47].

Compared with persons with influenza infection, those infected with HMPV had more underlying conditions, such as chronic respiratory disease, including COPD, CHF, and cardiovascular combined conditions, and were more likely to have received the influenza vaccination. That might be explained partially by the fact that 55% of HMPV-infected patients were 65 years or over, compared to 44% of influenza-infected patients, and the possibility of having comorbidities increases with age. The proportion requiring ICU admissions did not differ for HMPV and influenza. Similar results have been reported in previous studies [17, 19], including our previous study [22].

Our study had several limitations. Firstly, HMPV and influenza were not tested uniformly in all ARI hospitalized individuals, although multiple imputations were used as a correction method to improve estimates. However, the reason behind missingness remains an issue and should be considered when interpreting the results. Secondly, we may have missed counting some cases of both HMPV and influenza in non-SARI presentations outside of the winter season and also SARI cases, which changed from year round to winter seasons (May-October) for SARI and variable inclusion of non-SARI patients for testing. Surveillance in medical wards and ICUs was conducted year round from 2012 to 2015 and 2022 to 2023. During 2016 to 2021, ICUs were monitored year round, while general medical and pediatric wards were surveyed from May to October each year. Although most positive influenza cases were detected during winter, we may have dismissed some HMPV positives due to its delayed seasonality and lower testing rates compared to influenza. In addition, some morbidity caused by HMPV does not show up in acute respiratory wards. Our surveillance was limited in identifying influenza/HMPV virus-infected patients with atypical clinical presentations, which would result in an underestimation of the burden of influenza/HMPV hospitalization. Influenza/HMPV infection can lead to more severe illness and complications such as primary viral pneumonia, secondary bacterial pneumonia, cardiac complications, and neurological complications. Influenza/HMPV infection can also cause exacerbations of underlying diseases such as chronic lung disease or cardiovascular disease. Some complications and exacerbations may occur after typical influenza/HMPV-related clinical symptoms have resolved, and influenza/HMPV infection may not be suspected to cause these complications. Finally, without HMPV subtyping, we cannot detect which subgroup is predominant or associated with more severe disease.

## CONCLUSIONS

Although the HMPV infections accounted for fewer hospitalizations than influenza in adults aged 20 years and over, the difference in ARI hospitalization rates between young and old were more pronounced for HMPV than that of hospitalization rates associated with influenza. This underscores the need for antiviral therapy and vaccination against HMPV for this vulnerable group. Additionally, there are disproportionate ethnic and socioeconomic patterns in hospitalization rates for HMPV and influenza, which indicates a need for public health prioritization by health care systems and for pharmaceutical interventions. While no statistically significant increases in HMPV-associated hospitalization rates were observed following the COVID-19 pandemic, there was significant aggravation of ethnic and SES inequities among influenza-associated hospitalizations in 2022 and 2023.

## Supplementary Material

jiaf150_Supplementary_Data

## Supplementary Data

Supplementary materials are available at *The Journal of Infectious Diseases* online (http://jid.oxfordjournals.org/). Supplementary materials consist of data provided by the author that are published to benefit the reader. The posted materials are not copyedited. The contents of all supplementary data are the sole responsibility of the authors. Questions or messages regarding errors should be addressed to the author.

## Notes

***Author contributions.*** Q. S. H., N. A., and T. W. contributed conceptualization and methodology of the study. R. S., J. L., C. W., A. T., and C. G. actively contributed to the operation, testing, and meta-data collection used in this analysis. N. A., T. W., and L. T. did the data analysis. N. A. and B. F. wrote the first draft of the manuscript. All authors contributed to the interpretation of the results, revising the manuscript critically for intellectual content, and gave final approval of the version to be published.

SHIVERS investigation team: Institute of Environmental Science and Research: C. E. Tan, J. Bocacao, W. Gunn, J. Ralston, B. Bird, T. Slater, A. Farjana, K. Lodder, G. Hooper, C. Tarring, S. Utekar, M. A. Sam, A. Anglemyer, S. Jefferies, A. McNeill, G. Graham, L. Kelsall, B. Waite; Te Whatu Ora—Health New Zealand: Counties Manukau: S. Lawrence, S. Taylor, G. Spence, M. Maminta, R. Clark, A. Retter; and Te Whatu Ora—Health New Zealand: Auckland: A. Stanley, G. McAuliffe, S. Roberts, R. Westbury, K. Yorston, C. Sherring, S. Coetzer.

***Acknowledgments.*** SHIVERS SARI surveillance, led by the Institute of Environmental Science and Research (ESR), is a multicenter and multidisciplinary collaboration. The authors thank SHIVERS collaborating organizations for their commitment and support: ESR, University of Auckland, University of Otago, Auckland District Health Board (DHB) (now known as Te Whatu Ora—Health New Zealand Te Toka Tumai Auckland), Counties Manukau DHB (now known as Te Whatu Ora—Health New Zealand Te Toka Tumai Counties Manukau), WHO Collaborating Centre at St Jude Children's Research Hospital in Memphis, United States; participating virology laboratories in Auckland City Hospital, Middlemore Hospital, and ESR's WHO National Influenza Centre.

Disclaimer. The funding source has no role in study design, collection, analysis, or interpretation of data; writing of reports; or decision to submit papers for publication.

***Financial support.*** This work was supported by Icosavax, a member of the AstraZeneca Group. The SHIVERS SARI surveillance was funded by the US Centers for Disease Control and Prevention (grant number U01IP000480) during 2012–2016 and by the New Zealand Ministry of Health during 2017–2023.

***Supplement sponsorship.*** This article appears as part of the supplement “Human Metapneumovirus Disease Burden in Older Adults and Implications for Adult Vaccination Strategies,” sponsored by AstraZeneca.
