# Supplementary material for: The Burden of HMPV- and Influenza-Associated Hospitalizations in Adults in New Zealand Before and After the COVID-19 Pandemic, 2012–2023
Source: J Infect Dis. 2025 Jul 16;232(Suppl 1):S47–58. doi: 10.1093/infdis/jiaf150 (PMC12265057; doi:10.1093/infdis/jiaf150)
Supplement: jiaf150_Supplementary_Data [file jiaf150_supplementary_data.zip › Supplementary.docx]

**Supplementary Material A.**

**Figure S1. SHIVERS study enrolment status for adults aged ≥20 years hospitalised with acute respiratory infection (ARI) stratified by severe acute respiratory infection (SARI) and testing status for HMPV and Influenza, Auckland, New Zealand in 2012-2023**

37185 ARI * hospitalisations

by adult (aged 20+ years) residents of study hospital catchment area 2012-2023

2501 influenza+ 455 HMPV +

1122 influenza+ 280 HMPV+

14639

SARI** cases

22546

Non-SARI *** cases

10601 tested for influenza

10383 tested for HMPV

13537 tested for influenza

 11266 tested for HMPV

Not tested

Non-SARI cases

Not tested

SARI cases

61181 ARI * hospitalisations among

residents of study hospital catchment area

2012-2023

* ARI, acute respiratory infection

** SARI, severe ARI, defined as ARI with a history of fever or measured fever ≥ 38^◦^C and cough with onset in the preceding 10 days

*** Non-SARI non-SARI respiratory patients with either cough or measured/reported fever within 10 days

| **Table S1. Acute respiratory infections (ARIs) hospitalisations associated with HMPV or influenza among adults by year, Auckland sub-region and demographic characteristic, Auckland, New Zealand 2012-2023** | | | | | |  |
| --- | --- | --- | --- | --- | --- | --- |
| **Characteristics** | **ARI hospitalisations** | | | | |  |
|  | **Total** | **Tested** | | **Confirmed** | |  |
|  |  | ***HMPV*** | ***Influenza*** | ***HMPV*** | ***Influenza*** |  |
|  | **n** | **n** | **n** | **n (% of tested)** | **n (% of tested)** |  |
| **Overall** | 37185 | 21649 | 24138 | 735 (3.4) | 3623 (15.0) |  |
| **Year** |  |  |  |  |  |  |
| **2012** | 3482 | 1525 | 1366 | 67 (4.4) | 311 (22.8) |  |
| **2013** | 3748 | 1812 | 1171 | 64 (3.5) | 203 (17.3) |  |
| **2014** | 3492 | 2189 | 2121 | 92 (4.2) | 439 (20.7) |  |
| **2015** | 3926 | 2501 | 2468 | 87 (3.5) | 330 (13.4) |  |
| **2016** | 2970 | 1695 | 1562 | 70 (4.1) | 178 (11.4) |  |
| **2017** | 2346 | 1798 | 1633 | 48 (2.7) | 401 (24.6) |  |
| **2018** | 1709 | 1261 | 1202 | 70 (5.6) | 254 (21.1) |  |
| **2019** | 2524 | 2088 | 2094 | 64 (3.1) | 425 (20.3) |  |
| **2020** | 1718 | 976 | 1476 | 2 (0.2) | 4 (0.3) |  |
| **2021** | 1885 | 819 | 1117 | 6 (0.7) | 0 (0.0) |  |
| **2022** | 4114 | 1974 | 3000 | 71 (3.6) | 438 (14.6) |  |
| **2023** | 5271 | 3011 | 4928 | 94 (3.1) | 640 (13.0) |  |
| **Age in years** |  |  |  |  |  |  |
| **20-49** | 7853 | 4968 | 5442 | 159 (3.2) | 1,123 (20.6) |  |
| **50-64** | 8621 | 5241 | 5774 | 178 (3.4) | 915 (15.8) |  |
| **65-79** | 12032 | 6853 | 7746 | 253 (3.7) | 934 (12.1) |  |
| **80+** | 8679 | 4587 | 5176 | 145 (3.2) | 651 (12.6) |  |
| **Sex** |  |  |  |  |  |  |
| **Female** | 19362 | 11427 | 12708 | 454 (4.0) | 2,072 (16.3) |  |
| **Male** | 17766 | 10200 | 11404 | 281 (2.8) | 1,547 (13.6) |  |
| **Ethnicity** |  |  |  |  |  |  |
| **European & other** | 16387 | 9303 | 10143 | 335 (3.6) | 1340 (13.2) |  |
| **Māori** | 6088 | 3504 | 4042 | 79 (2.3) | 518 (12.8) |  |
| **Pacific people** | 10201 | 6080 | 6929 | 232 (3.8) | 1255 (18.1) |  |
| **Asians** | 4113 | 2514 | 2784 | 81 (3.2) | 470 (16.9) |  |
| **SES**** |  |  |  |  |  |  |
| **1 (least deprived)** | 3456 | 1970 | 2162 | 71 (3.6) | 320 (14.8) |  |
| **2** | 5676 | 3254 | 3540 | 122 (3.7) | 518 (14.6) |  |
| **3** | 5846 | 3380 | 3680 | 114 (3.4) | 516 (14) |  |
| **4** | 6621 | 3861 | 4435 | 116 (3) | 631 (14.2) |  |
| **5 (most deprived)** | 15238 | 9013 | 10111 | 310 (3.4) | 1603 (15.9) |  |

*Socioeconomic status (SES) based on small area-level measure of household deprivation derived from the national census (NZ Dep 2013) where 1 indicates the individual is living in a household that is in the least socioeconomic-deprived quintile.

| **Table S2. Comparison of** **characteristics of acute respiratory hospitalisations (ARIs) among adults across HMPV and influenza testing status, Auckland, New Zealand 2012-2023** | | | | | | | | |  |  |
| --- | --- | --- | --- | --- | --- | --- | --- | --- | --- | --- |
|  |  |  |  |  |  |  |  |  |  | |
| **Characteristics** | **Number of ARI events** | **Tested for HMPV n (% row)** | | |  | **Tested for Influenza n (% row)** | | |  | |
|  |  | **overall** | **SARI** | **Non-SARI** |  | **overall** | **SARI** | **Non-SARI** |  | |
| **Overall** | 37185 | 21649 (58.2) | 10383 (48) | 11266 (52) |  | 24138 (64.9) | 10601 (43.9) | 13537 (56.1) |  | |
| **Year** |  |  |  |  |  |  |  |  |  | |
| 2012 | 3482 | 1525 (43.8) | 1185 (77.7) | 340 (22.3) |  | 1366 (39.2) | 1069 (78.3) | 297 (21.7) |  | |
| 2013 | 3748 | 1812 (48.3) | 1320 (72.8) | 492 (27.2) |  | 1171 (31.2) | 739 (63.1) | 432 (36.9) |  | |
| 2014 | 3492 | 2189 (62.7) | 1221 (55.8) | 968 (44.2) |  | 2121 (60.7) | 1175 (55.4) | 946 (44.6) |  | |
| 2015 | 3926 | 2501 (63.7) | 1063 (42.5) | 1438 (57.5) |  | 2468 (62.9) | 1057 (42.8) | 1411 (57.2) |  | |
| 2016 | 2970 | 1695 (57.1) | 647 (38.2) | 1048 (61.8) |  | 1562 (52.6) | 615 (39.4) | 947 (60.6) |  | |
| 2017 | 2346 | 1798 (76.6) | 1048 (58.3) | 750 (41.7) |  | 1633 (69.6) | 904 (55.4) | 729 (44.6) |  | |
| 2018 | 1709 | 1261 (73.8) | 798 (63.3) | 463 (36.7) |  | 1202 (70.3) | 746 (62.1) | 456 (37.9) |  | |
| 2019 | 2524 | 2088 (82.7) | 857 (41) | 1231 (59) |  | 2094 (83) | 863 (41.2) | 1231 (58.8) |  | |
| 2020 | 1718 | 976 (56.8) | 314 (32.2) | 662 (67.8) |  | 1476 (85.9) | 409 (27.7) | 1067 (72.3) |  | |
| 2021 | 1885 | 819 (43.4) | 260 (31.7) | 559 (68.3) |  | 1117 (59.3) | 351 (31.4) | 766 (68.6) |  | |
| 2022 | 4114 | 1974 (48) | 648 (32.8) | 1326 (67.2) |  | 3000 (72.9) | 1010 (33.7) | 1990 (66.3) |  | |
| 2023 | 5271 | 3011 (57.1) | 1022 (33.9) | 1989 (66.1) |  | 4928 (93.5) | 1663 (33.7) | 3265 (66.3) |  | |
| **Age (years)** |  |  |  |  |  |  |  |  |  | |
| 20-49 | 7853 | 4968 (63.3) | 2887 (58.1) | 2081 (41.9) |  | 5442 (69.3) | 2970 (54.6) | 2472 (45.4) |  | |
| 50-64 | 8621 | 5241 (60.8) | 2618 (50) | 2623 (50) |  | 5774 (67) | 2659 (46.1) | 3115 (53.9) |  | |
| 65-79 | 12032 | 6853 (57) | 2990 (43.6) | 3863 (56.4) |  | 7746 (64.4) | 3087 (39.9) | 4659 (60.1) |  | |
| 80+ | 8679 | 4587 (52.9) | 1888 (41.2) | 2699 (58.8) |  | 5176 (59.6) | 1885 (36.4) | 3291 (63.6) |  | |
| **Sex*** |  |  |  |  |  |  |  |  |  | |
| Female | 19362 | 11427 (59) | 5952 (52.1) | 5954 (52.1) |  | 12708 (65.6) | 5573 (43.9) | 7135 (56.1) |  | |
| Male | 17766 | 10200 (57.4) | 5306 (52) | 5312 (52.1) |  | 11404 (64.2) | 5010 (43.9) | 6394 (56.1) |  | |
| **Ethnicity*** |  |  |  |  |  |  |  |  |  | |
| European & other | 16387 | 9303 (56.8) | 4066 (43.7) | 5237 (56.3) |  | 10143 (61.9) | 4056 (40) | 6087 (60) |  | |
| Māori | 6088 | 3504 (57.6) | 1629 (46.5) | 1875 (53.5) |  | 4042 (66.4) | 1704 (42.2) | 2338 (57.8) |  | |
| Pacific people | 10201 | 6080 (59.6) | 3255 (53.5) | 2825 (46.5) |  | 6929 (67.9) | 3405 (49.1) | 3524 (50.9) |  | |
| Asians | 4113 | 2514 (61.1) | 1307 (52) | 1207 (48) |  | 2784 (67.7) | 1324 (47.6) | 1460 (52.4) |  | |
| **SES*** |  |  |  |  |  |  |  |  |  | |
| Highest quintile ¥ | 3456 | 1970 (57) | 967 (49.1) | 1003 (50.9) |  | 2162 (62.6) | 971 (44.9) | 1191 (55.1) |  | |
| 2 | 5676 | 3254 (57.3) | 1481 (45.5) | 1773 (54.5) |  | 3540 (62.4) | 1519 (42.9) | 2021 (57.1) |  | |
| 3 | 5846 | 3380 (57.8) | 1558 (46.1) | 1822 (53.9) |  | 3680 (62.9) | 1555 (42.3) | 2125 (57.7) |  | |
| 4 | 6621 | 3861 (58.3) | 1759 (45.6) | 2102 (54.4) |  | 4435 (67) | 1846 (41.6) | 2589 (58.4) |  | |
| Lowest quintile | 15238 | 9013 (59.1) | 4525 (50.2) | 4488 (49.8) |  | 10111 (66.4) | 4607 (45.6) | 5504 (54.4) |  | |
| ¥ Socioeconomic status (SES) based on small area-level measure of household deprivation derived from the national census (NZ Dep 2013, 2018) | | | | | | | | |  |  |
| *Total numbers may not be the same due to missing values | | | | | | | | |  |  |

**Supplementary Material B.**

**The Charlson Comorbidity Index**

In this report, using the ICD10 codes, we calculated the Charlson Comorbidity Index (CCI).

The term comorbidity has a Latin origin and results from the combination of two words: "co," meaning "along with," and "morbus," meaning "disease." It was Alvan R. Feinstein who provided the first clinical definition of this concept, which refers to "any distinct additional clinical entity that has existed or that may occur during the clinical course of a disease that is under study" (1). Further, he noted that a comorbid condition has the potential to impact a patient's prognosis and alter therapeutic plans and outcomes (1).

The CCI, developed in 1987, became the most widely used index and is often considered the gold-standard measure for assessing comorbidity in clinical research (2).

Multimorbidity is commonly understood to be the coexistence of multiple health conditions in an individual (3,4). It is a growing global public health challenge as populations ageing and the prevalence of long-term conditions rises (3,4). A related term, comorbidity, describes the burden of illness coexisting with a particular disease of interest (5).

The CCI compiles International Classification of Disease (ICD) codes for diseases such as myocardial infarction, congestive heart failure, peripheral vascular disease, cerebrovascular disease, pulmonary diseases, rheumatic disease, dementia, hemiplegia, diabetes, chronic kidney disease, liver disease, peptic ulcer disease, cancer, and HIV/AIDS. Over time, the CCI has gained traction as a reliable measure of comorbidity burden and is routinely used as a covariate in regression models.

The CCI, a comprehensive scoring system, assigns different scores for different comorbidities. This includes 1 point for each myocardial infarction, congestive heart failure, peripheral vascular disease, dementia, cerebrovascular disease, chronic lung disease, connective tissue disease, ulcer, chronic liver disease, and diabetes. Similarly, it assigns 2 points for plegia, moderate or severe kidney disease, diabetes with end-organ damage, tumor, leukaemia, and lymphoma. The system further distinguishes with 3 points for moderate or severe liver disease and a significant 6 points for tumor metastasis or AIDS.

Over time, the CCI has been modified, revised, and simplified, with some versions receiving their names (Charlson/Deyo (6), Charlson/Romano (7), Charlson/Manitoba (8), Charlson/D'Hoores (9), Charlson/Ghali (10), and Charlson/ Quan (11). These versions often use different models based on the weighting of comorbidities (12). For example, Quan et al. (11) also included leukaemia and lymphoma among malignancies and expanded "hemiplegia" to "paresis/plegia," encompassing conditions that affect symmetrical parts and the entire body.

**Supplementary Table S3. Classical and updated Charlson comorbidity index weights**

| Charlson comorbidities | Updated CCI | Original CCI* |
| --- | --- | --- |
| Myocardial infarction | 0 | 1 |
| Congestive heart failure | 2 | 1 |
| Peripheral vascular disease | 0 | 1 |
| Cerebrovascular disease | 0 | 1 |
| Dementia | 2 | 1 |
| Chronic pulmonary disease | 1 | 1 |
| Rheumatologic disease | 1 | 1 |
| Peptic ulcer disease | 0 | 1 |
| Mild liver disease | 2 | 1 |
| Diabetes without chronic complications | 0 | 1 |
| Diabetes with chronic complications | 1 | 2 |
| Hemiplegia or paraplegia | 2 | 2 |
| Renal disease | 1 | 2 |
| Any malignancy, including leukaemia and lymphoma | 2 | 2 |
| Moderate or severe liver disease | 4 | 3 |
| Metastatic solid tumor | 6 | 6 |
| AIDS/HIV | 4 | 6 |
| Maximum comorbidity score | 24 | 29 |

* The Charlson Comorbidity Index (CCI)

**References:**

1. Feinstein AR. The pre-therapeutic classification of co-morbidity in chronic disease. J Chronic Dis. 1970 Dec;23(7):455–68.

2. Charlson ME, Pompei P, Ales KL, MacKenzie CR. A new method of classifying prognostic comorbidity in longitudinal studies: development and validation. J Chronic Dis. 1987 Jan;40(5):373–83.

3. Uijen AA, Van de Lisdon EH. Multimorbidity in primary care: prevalence and trend over the last 20 years. Eur J Gen Pract 2008;14:28–32.

4. Fortin M, Stewart M, Poitras M, et al. A systematic review of prevalence studies on multimorbidity: toward a more uniform methodology. Ann Fam Med 2012;10:142–51.

5. Leal JR, Laupland KB. Validity of ascertainment of co-morbid illness using administrative databases: a systematic review. Clin Microbiol Infect 2010;16:715–21.

6- Deyo RA, Cherkin DC, Ciol MA. Adapting a clinical comorbidity index for use with ICD-9-CM administrative databases. J Clin Epidemiol. 1992;45(6):613–619. doi:10.1016/0895-4356(92)90133-8

7. Romano PS, Roos LL, Jollis JG. Adapting a clinical comorbidity index for use with ICD-9-CM administrative data: differing perspectives. J Clin Epidemiol. 1993;46(10):1075–9;discussion 81–90. doi:10.1016/0895-4356(93)90103-8

8. Roos LL, Sharp SM, Cohen MM, et al. Risk adjustment in claims- based research: the search for efficient approaches. J Clin Epidemiol. 1989;42(12):1193–1206. doi:10.1016/0895-4356(89)90118-2

9.D’Hoore W, Sicotte C, Tilquin C. Risk adjustment in outcome assess-ment: the Charlson comorbidity index. Methods Inf Med. 1993;32 (5):382–387.

10. Ghali WA, Hall RE, Rosen AK, et al. Searching for an improved clinical comorbidity index for use with ICD-9-CM administrative data. J Clin Epidemiol. 1996;49(3):273–278. doi:10.1016/0895-4356 (95)00564-1

11. Quan H, Sundararajan V, Halfon P, et al. Coding algorithms for defining comorbidities in ICD-9-CM and ICD-10 administrative data. Med Care. 2005;43(11):1130–1139. doi:10.1097/01. mlr.0000182534.19832.83

12. Yurkovich M, Avina-Zubieta JA, Thomas J, et al. A systematic review identifies valid comorbidity indices derived from administrative health data. J Clin Epidemiol. 2015;68(1):3–14. doi:10.1016/j. jclinepi.2014.09.01013

**Supplementary Material C.**

**‘Suspected acute respiratory infection’ definition**

Research nurses conducted weekday assessment (and retrospective weekend day assessment) of inpatients with suspected respiratory infections or febrile illness of unknown source to establish whether each met the WHO SARI case definition or not. Prior to assessment against the SARI case definition, ‘suspected ARI’ inpatients were identified based on presentation with the following broad conditions:

- Suspected acute upper respiratory tract infection (including coryza, pharyngitis)
- Suspected croup
- Suspected pneumonia
- Exacerbations of asthma
- Exacerbations of adult chronic lung disease (including COPD, emphysema, bronchitis)
- Respiratory failure
- Febrile illness with respiratory symptoms (including shortness of breath)
- Other suspected acute respiratory infections

Broad respiratory conditions that were not considered ‘suspected ARI’ included:

- Other diseases of the upper respiratory tract e.g. allergic rhinitis
- Lung diseases due to external agents e.g. pneumonitis
- Other respiratory diseases principally affecting the interstitium e.g. Pulmonary oedema
- Suppurative and necrotic conditions of the lower respiratory tract e.g. Abscess of lung and mediastinum
- Diseases of pleura e.g. Pleural effusion not elsewhere classified, Pneumothorax
- Other diseases of the respiratory system e.g. Postprocedural respiratory disorders, not elsewhere classified

For all cases meeting the SARI case definition, an electronic record was created in the surveillance project. For adult cases that did not meet the SARI case definition criteria (i.e. non-SARI cases), electronic surveillance project records were created for a subset of patients only, with non-SARI quotas until study year 2017 and then inconsistent ward-specific non-SARI case reporting practices in subsequent years. However, non-SARI cases admitted to ICU wards were consistently reported into the surveillance project in all years across all ages.
